# Comparison of changes in retinal nerve fiber layer thickness and intraocular pressure between glaucoma and non-glaucoma patients after phacoemulsification

**DOI:** 10.12669/pjms.39.1.6531

**Published:** 2023

**Authors:** Maryam Shahid, Zubair Saleem, Tayyaba Gul Malik, Muhammad Farqaleet

**Affiliations:** 1 Dr. Maryam Shahid, Ophthalmology Department, Lahore General Hospital, Lahore, Pakistan; 2 Dr. Zubair Saleem, Ophthalmology Department, Lahore General Hospital, Lahore, Pakistan; 3 Prof. Tayyaba Gul Malik, Ophthalmology Department, Lahore General Hospital, Lahore, Pakistan; 4 Dr. Muhammad Farqaleet, Ophthalmology Department, Lahore General Hospital, Lahore, Pakistan

**Keywords:** Glaucoma, Cataract, Retinal Nerve Fiber Layer, Optical Coherence Tomography, Intraocular pressure

## Abstract

**Objective:**

To compare the changes in Retinal Nerve Fiber Layer (RNFL) thickness and Intraocular Pressure (IOP) after phacoemulsification in patients with glaucoma versus without glaucoma.

**Methods:**

This was a quasi-experimental study, done at Lahore General Hospital, from January 2021 to December 2021. Patients with senile cataract were divided into two groups. One group included 40 patients, with ≥40 years of age and diagnosed with primary open angle glaucoma compared with a second group of 40 age-matched controls without glaucoma, undergoing phacoemulsification and Intraocular Lens Implantation (IOL). Goldman Applanation Tonometer (GAT) and Spectral Domain Optical Coherence Tomography (SD-OCT) were used to record IOP and RNFL thickness, before surgery, at one week and one month after surgery. The results were compared with baseline readings and also between the two groups.

**Results:**

A total of 80 patients were included in the study. Mean age was 60.3±7.9 years with male to female ratio of 1:1. Mean change in RNFL thickness in glaucomatous eyes group and non-glaucomatous Eyes group from pre-operative baseline was 11.33±4.30μm and 4.08±2.59μm respectively after one month (p-value<0.001). Difference of mean change in RNFL thickness from baseline was statistically significant between both groups (p< 0.001). Difference of mean change in IOP from baseline at four weeks was statistically significant in the individual groups but between both groups was statistically insignificant (p= 0.234)

**Conclusion:**

Phacoemulsification results in increase in RNFL thickness and decrease in IOP, which are good prognostic factors in control of glaucoma. However, a mean change of IOP of 1mmHg in glaucoma patient does not affect management of glaucoma.

## INTRODUCTION

A common clinical feature in glaucoma patients is progressive decrease in Retinal Nerve Fiber Layer (RNFL) thickness. Changes in RNFL thickness have been used not only for the diagnosis but for monitoring of disease progression as well. Among the various imaging modalities, Optical Coherence Tomography (OCT) has gained lot of interest in the assessment of RNFL thickness due to its convenience, accuracy and minimal inter-observer variability.^1^,^2^

Existing literature indicates that phacoemulsification results in improvement of RNFL thickness and decrease in Intraocular Pressure (IOP). Jha et al. used spectral domain OCT for assessment of retinal changes following cataract surgery and reported that there was increase in mean RNFL thickness one week after the surgery.^3^ Studies have also shown that there is reduction of IOP probably owing to widening of anterior chamber depth and increased drainage of aqueous humor.^4^ However, literature either shows effect of cataract surgery on IOP and RNFL thickness in patients with cataract alone or if they included glaucoma patients, the comparison between the glaucoma and non-glaucoma was not made. One research compared RNFL thickness between glaucomatous and non-glaucomatous eyes.^5^ However, the available research evidence was limited and there was no such published research in the local population, which necessitated the conduction of present study. As RNFL thickness is important marker in the diagnosis, prognosis and risk stratification of patients with glaucoma, so there is need to study the effect of cataract surgery in patients with co-existing glaucoma and to compare the results with non-glaucomatous eyes. Only those cataract patients were included whose OCT showed reliable RNFL values indicated by good signal strength

The study would help find out the effects of phacoemulsification on RNFL thickness of glaucoma and non-glaucoma patients and the knowledge will be beneficial for patients’ treatment in future. The use of phacoemulsification may alone improve RNFL thickness and may reduce the need of multiple anti glaucoma medicines.

## METHODS

It was a quasi-experimental study, conducted at Department of Ophthalmology, Lahore General Hospital, Lahore. Duration of study was 12 months, from January 2021 to December 2021. The sample size of 40 in each group was calculated by keeping the power of study equal to 80% and level of significance equal to 5%.^5^

Patients were selected by non-probability, purposive sampling. Approval for the study was sought from the Institutional Review Board of hospital (Reference No: 00-209-20; dated 16 November 2020). An informed consent was obtained from every patient for enrollment in the study. Patients > 40 years of age requiring cataract surgery, with nuclear sclerosis of grades 1-2 according to Lens Opacification Classification System III and having IOP less than 21mm Hg were included. The patients were divided into two groups. In one group, patients with cataract and Primary Open Angle Glaucoma (POAG) controlled on medication (either single drug or multiple) were included and second group included non-glaucoma patients with cataract. Signal strength of 5/10 and above was considered acceptable.

Eyes with lens changes other than senile cataract, macular diseases which cause changes in macular thickness, history of previous ocular surgery/trauma, dense lenticular changes affecting the signal to noise ratio, media opacity and optic disc anomalies were excluded.

All patients underwent complete history and ocular examination including Goldmann Applanation tonometry, Fundoscopy and spectral domain optical coherence tomography (SD-OCT) after pupillary dilatation using Tropicamide 1% eye drops. Three scans were obtained and the scan with best signal strength was included in the study. Peri-papillary retinal nerve fiber layer (RNFL) scan (4 quadrant scan) was used to measure the average RNFL thickness. Scans with poor reliability were rejected, and repeat scans were acquired. Patients in both groups underwent phacoemulsification with intra ocular lens (IOL) implantation under local anesthesia by an experienced surgeon (with more than five years’ experience of phacoemulsification). Drug regimen included topical antibiotic-steroid combination for both groups. Postoperative OCT and IOP were checked on each follow-up visit and RNFL thickness was recorded. OCT and IOP were repeated at one week and one month after surgery.

Data was analyzed using SPSS version 23.0. Numerical variables; age, pre- and post-operative RNFL thickness and change in RNFL thickness, IOP, change in IOP were described in terms of mean±SD. Categorical variable; gender was described in terms of frequency and percentage. Both the groups were compared for mean pre- and post-operative IOP, mean change in IOP, RNFL thickness and mean change in RNFL thickness using t-test. P-value of ≤ 0.05 was considered statistically significant.

## RESULTS

Age range of patients was 40-86 years (mean 60.3±7.9 years). Twenty-seven (33.8%) patients were less than 60 years and 53 (66.2%) were 60 years and above. There were 40 (50.0%) males and 40 (50.0%) females. Mean age was 60.3±7.9 years. Both the groups were comparable in terms of mean of age (p-value=0.911) and likewise distribution of various subgroups based on age (p-value=0.813) and gender (p-value=0.655).

RNFL thickness decreased in both groups after first week and a greater change was observed in non-glaucomatous eyes as compared to glaucomatous eyes. The difference between the groups was statistically insignificant (p-value=0.113). At one month, there was a significant increase in mean RNFL thickness in both groups when compared with baseline (p <0.001). When compared between the two groups, mean change in RNFL thickness after one month was significantly greater in glaucomatous eyes as compared to non-glaucomatous eyes (p < 0.001).

Regarding IOP, there was statistically significant increase in the intraocular pressure in each group at one week after the surgery. After one month, there was statistically significant reduction in the mean IOP in each group from the baseline (p < 0.05). Mean change in IOP between the two groups was statistically insignificant (p =0.234) Table-I.

**Table-I:** Retinal Nerve Fiber Layer Thickness (μm) and intra ocular pressure at Baseline, 1 week and 1 Month after the Surgery.

	RNFL Thickness (μm)		P-value	Intraocular Pressure (mmHg)		P-value
	
	Glaucomatous Eyes n=40	Non-Glaucomatous Eyes n=40		Glaucomatous Eyes n=40	Non-Glaucomatous Eyes n=40	
Pre-Operatively	74.57±7.99	94.68±4.64	<0.001*	12.40±1.22	13.35±1.59	0.004*
1 Week Post-Operatively	74.27±7.59	91.87±7.10	<0.001*	15.80±3.16	15.05±3.17	0.293
Change from Baseline	0.30±8.58	2.80±4.87	0.113	3.40±3.15	1.70±3.35	0.022*
P-value	0.012~	<0.001~		<0.001~	0.003~	
1 Month Post-Operatively	85.90±5.44	98.75±4.41	<0.001*	11.70±1.16	12.20±1.35	0.079
Change from Baseline	11.33±4.30	4.08±2.59	<0.001*	0.70±1.54	1.15±1.81	0.234
P-value	<0.001~	<0.001~		0.006~	<0.001~	

The observed difference was statistically significant on

* Independent sample t-test for comparison of RNFL thickness between the groups

~ Paired sample t-test for intragroup comparison of RNFL thickness from baseline at one month follow-up.

## DISCUSSION

The results of this study showed that following an initial decrease in RNFL thickness after 1^st^ post-operative week of phacoemulsification, there was a subsequent increase in RNFL thickness at one month in both groups. However, the increase in RNFL thickness was significantly higher in glaucomatous eyes as compared to the non-glaucomatous eyes indicating a beneficial effect of phacoemulsification in Glaucoma. However, long-term assessment of RNFL thickness is required. A similar but inverse relationship was observed in intraocular pressure, which increased initially at week one and decreased at one month after surgery. This also indicated that phacoemulsification has an additional advantage of IOP reduction although the decrease in IOP was not significant.

Similar changes in IOP and mean RNFL thickness were observed by Abdelghani et al. who suggested that cataract surgery might alone be enough to control or help in controlling IOP among pseudo-exfoliative glaucoma patients and alleviate the need for any additional surgery for this type of glaucoma.^6^

It has been reported that changes in IOP and tissue damage with subsequent inflammation cause transient macular edema appearing as an increased macular thickness immediately after cataract surgery. The increase in macular thickness is also associated with an increase in RNFL thickness over long-term follow-up.^7^ Another study showed that phacoemulsification resulted in increase of RNFL thickness.^3^ The authors observed that mean RNFL thickness increased from 92.6±5.4μm to 101.3±5.6μm one month after surgery. Similar change in RNFL thickness was reported by Nasar et al.^8^ Contrary to this, another study compared the effects of Femto laser assisted cataract surgery (FLACS) and conventional phacoemulsification on RNFL thickness. It was concluded that both procedures resulted in RNFL thinning without any statistically significant difference between the two procedures.^9^ In a Korean research on glaucomatous eyes undergoing phacoemulsification it was observed that mean RNFL thickness increased from 83.41±12.29μ to 94.14±10.72μm one month after the surgery and persisted until three months.^10^ The immediate increase in RNFL thickness can be explained by post-operative edema but persistent increase one month after the surgery is clinically significant as it indicates substantial benefit of phacoemulsification for glaucoma, which has been seen in our results.

Perdana et al. compared changes in mean RNFL thickness after phacoemulsification in patients with glaucoma versus without glaucoma. The authors reported that there was increase in RNFL thickness in both the groups.^5^ However, the mean change was significantly higher in glaucomatous eyes (p-value<0.001) as compared to non-glaucomatous eyes. The results were consistent with our study but their sample size was only 26 patients. They also observed visual fields deficits in glaucomatous eyes, which were not considered in our study.

Our study included patients with controlled open angle glaucoma whereas, other recent studies have shown that phacoemulsification resulted in increase in anterior chamber depth and decrease in IOP in Primary angle closure and other spectrum of angle closure diseases.^11^,^12^

Certain parameters which affect the decrease in IOP were studied by Lin C et al and iris thickness and lens vault were found to be associated with decrease in IOP.^13^ When changes in anterior chamber angle which lead to decrease in IOP were compared between eyes with narrow angle and normal eyes, it was seen that these changes never reached the eyes of normal values.^14^ Zuo et al included patients with medically uncontrolled filtered Primary angle closure glaucoma. They reported decrease IOP and improved vision in these patients.^15^

Our study showed an initial increase and a later decease in IOP at one month in glaucomatous as well as non-glaucomatous eyes. However, Majstruk reported a modest decrease in IOP even at one year after surgery.^16^ In his study, IOP remained unstable during the first post-operative month and reduced within three months after surgery.

Literature shows that there is a more significant change in IOP within one week after surgery in emmetropes and mild to moderate myopes. However, significant decrease in IOP is found in high myopes during second and third post-operative months.^17^ Patients older than 75 years and female patients had more effect on reduction of IOP.^17^ We did not consider these parameters in this particular study. Decrease in IOP was seen during first three months in a local study as well.^18^

In our study, we excluded patients with pseudo-exfoliation. However, it was reported by Ramezani et al. that there was a greater reduction in IOP in eyes with pseudo-exfoliation as compared to the controls.^19^ Predictors for reduction in IOP were studied by Claudio et al. They found that preoperative IOP, anterior chamber depth (ACD), IOP/ACD ratio and gonioscopy score affected the post-operative IOP results.^20^

A possible mechanism for the beneficial effect on glaucoma seen in our study might be opening up of the phakic angle to wider pseudophakic angle resulting in increased drainage of aqueous humor and decrease IOP and improvement in RNFL thickness. It is important to remember that reduction in mean IOP following phacoemulsification may not meet IOP targets in glaucoma patients and additional IOP lowering medications and/or surgery might be required.

The present study gives results in local population and adds to the already published international research evidence on the topic. Our study advocates that cataract surgery has a moderate beneficial effect in the management of such patients with or without IOP-lowering medications and need for additional surgery for glaucoma should be carefully assessed. The strengths of the present study were its large sample size of 80 cases and strict exclusion criteria.

### Limitations:

It was its limited follow-up of one month. A long-term follow-up would have allowed us to track progression of glaucoma and need for definitive glaucoma surgery among such patients, which could have established the long-term effects of cataract surgery in glaucoma patients more precisely. Comparison of preoperative and post-operative angle was also not done in this study. Effect on the ganglion cell is also a parameter which needs to be studied in the future research. Such a study is imperative and is recommended in future clinical research.

## CONCLUSION

In patients of cataract with glaucoma, phacoemulsification may have a beneficial effect on IOP and RNFL thickness with or without anti-glaucoma medications. A step-wise approach should be followed in patients with cataract and glaucoma.

### Authors’ Contribution:

**MS** collected data, analyzed it, revised and finally approved the manuscript. **ZS** conceived idea, designed the project, revised and finally approved the manuscript. **TGM** Analyzed data, drafted manuscript and revised it and approved the manuscript. **MF** contributed to conception & design of study, revised and finally approved manuscript. All authors agree to be accountable for all aspects of the work in ensuring that questions related to the accuracy or integrity of any part of the work are appropriately investigated and resolved.
